# Renal Trauma: Case Reports and Overview

**DOI:** 10.1155/2012/207872

**Published:** 2012-11-11

**Authors:** Campbell D. Tait, B. K. Somani

**Affiliations:** ^1^Department of Urology, Aberdeen Royal Infirmary, Aberdeen 25 2ZN, UK; ^2^Department of Urology, University Hospitals Southampton NHS Trust, Southampton 16 6YD, UK

## Abstract

*Introduction*. Renal trauma patients are largely managed conservatively but on occasion have to be embolised or taken to theatre for definitive surgical management, usually in the form of emergency nephrectomy. *Review*. We present an overview of renal trauma as illustrated by three interesting cases of blunt renal trauma who presented in quick succession of each other to the Emergency Department. The first case—a 48-year-old-female passenger in a road traffic accident—was treated with life-saving emergency nephrectomy. The second patient—a 47-year-old man who sustained a high impact injury whilst sledging—was managed conservatively on HDU and subsequently on the urology ward. The third patient—an 18-year-old man involved in a road traffic accident—underwent selective embolisation of a pseudoaneurysm after conservative therapy. *Discussion*. This case series illustrates the surgical, radiological, and conservative approaches to the management of significant renal trauma, which is potentially life threatening.

## 1. Introduction 

Renal trauma patients are largely managed conservatively but on occasion have to be taken to theatre for definitive surgical management, or embolised in interventional radiology. These patients can demonstrate true urological emergency. In this paper we discuss renal trauma and its management, illustrated by three cases of blunt renal trauma managed differently. 

The kidney is the organ most commonly associated with urological trauma and is involved in 1–20% of trauma cases [1–3]. The severity of renal trauma can range significantly, and thus the management options likewise can vary. Nonoperative management has become more commonplace in recent times, with the advent of interventional radiology and improvements in imaging. Furthermore, this approach is based on a better understanding of the ability of the kidney to sustain such injury and evade surgical help to recover. However, emergency nephrectomy remains the gold standard treatment for acute uncontrollable renal haemorrhage [1, 2]. 

## 2. Grading 

The American Association for the Surgery of Trauma (AAST) has produced an organ severity scale for the grading of renal trauma, in accordance with extent of injury sustained [4]. This scale has been prospectively validated and is directly associated with the need for surgical management in renal trauma patients. (See Table 1).

The AAST has also been shown to correlate with effect on renal function and can thus be applied as a predictor of outcome. One study, which showed this, reported mean decrease in renal function of 15%, 30%, and 65% after grade III, IV, and V injuries, respectively. Furthermore this study found that loss of renal function is independent of whether the injury is blunt or penetrating and whether treatment was surgical repair or conservative [5]. 

## 3. Management 

As with all trauma cases, a systematic approach following the principles of Advanced Trauma Life Support (ATLS) should be applied—ensuring that airway, breathing, and circulatory dysfunction are assessed and treated appropriately in the first instance [6]. Haemodynamic instability which is not corrected with crystalloid and blood product resuscitation should alert the surgeon to ongoing visceral haemorrhage requiring intervention. CT scanning remains the gold standard investigation of renal trauma where patient stability allows. Angiography can be useful in localising vascular injury and thus helping to target intervention [1, 6]. 

Grade I and II renal injuries are always managed conservatively. This varies from rest and analgesia to monitoring of vital signs until normalisation and resolution of haematuria, with antibiotic cover where appropriate [1]. Historically, the surgical management of grade III renal injuries has been controversial, but current evidence favours the nonoperative approach [7]. A recent prospective, multicentre observational study suggests that conservative management of grade IV renal injuries in the majority of cases preserves renal function as measured by dimercaptosuccinic acid renal scintigraphy [8]. 

A systematic review and meta-analysis of nonoperative management of nonvascular grade IV paediatric renal trauma concluded that the nonoperative approach was highly successful, with partial renal preservation achieved in 95% of patients [10]. Around one quarter of Grade 4 renal injuries are managed conservatively [1]. The preservation of renal parenchyma and avoidance of morbidity are the chief goals, which are very much achievable in paediatric patients with stable, grade I–III renal injuries. Similar outcomes are achievable with grade IV injuries [10]. 

Grade V renal trauma injuries have a poor outcome in terms of renal function. It is suggested that further study is required to compare conservative treatment versus nephrectomy in high grade blunt renal injury [8]. Grade V injury and the need for platelet transfusion in addition to other ongoing blood product and fluid requirements predict that need for emergency intervention. Increasing age and hypotension at time of presentation have been found to predict complications [11]. A recent 10 year retrospective review from an emergency hospital reported an emergency nephrectomy rate of over 80% for grade V renal injuries [3]. Conservative management has been found to be more commonplace at level I (most acute) trauma centres, and improved outcomes are achieved amongst more severely injured patients treated at level I centres [12]. 

Surgery must be performed on those patients who remain haemodynamically unstable despite resuscitation with crystalloid and blood products, with a likely renal haemorrhage and expanding retroperitoneal haematoma. Ideally, imaging with contrast CT or intravenous pyelogram should take place pre-operatively, although patients with Grade 5 renal injuries may not always be stable enough to undergo such investigation [1]. 

Penetrating injuries in particular have traditionally been managed with surgical intervention in the past, given the higher likelihood of life threatening injury. However, stab wounds and gunshot wounds to the kidney can be managed successfully without surgical intervention [13]. 

As well as complete nephrectomy, renal reconstruction and salvage can be possible where proximal vascular control is gained early, and haemorrhage is stemmed. Renorrhaphy or partial nephrectomy may be the most appropriate form of surgical intervention in cases of major polar injuries or mid-renal lacerations respectively. Approach in these cases is via midline laparotomy, allowing adequate assessment of other viscera. Reconstruction is based on principles of exposure, haemorrhage control, debridement and haemostasis. A watertight collecting system is paramount, and retroperitoneal drainage is important [13]. 

A low threshold for repeat CT scanning in the case of the patient who remains persistently tachycardic and anaemic with ongoing flank pain is required to aid diagnosis of continued or secondary bleeding due to pseudoaneurysm formation (as illustrated by Patient 3) or arteriovenous fistula formation. A falling haematocrit, or persistent pyrexia are also indications for repeat imaging [1]. 

Three interesting cases of high grade, blunt renal trauma are discussed below. They represent conservative, surgical and interventional approaches to the management of blunt renal trauma. Each patient was managed with a different approach, and all three made excellent recoveries. 

## 4. Patient 1 

**Figure 1:**
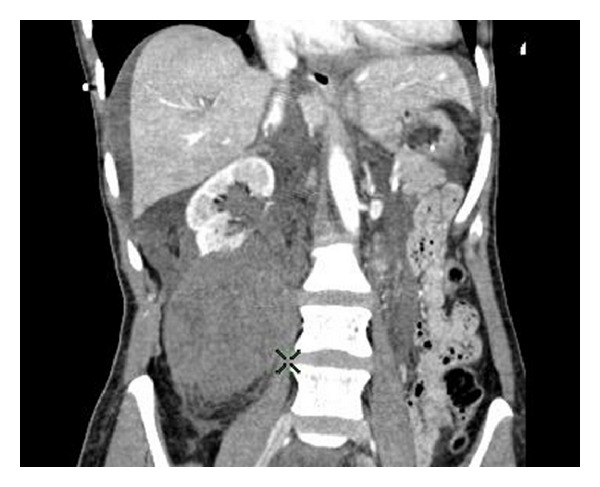


**Figure 2:**
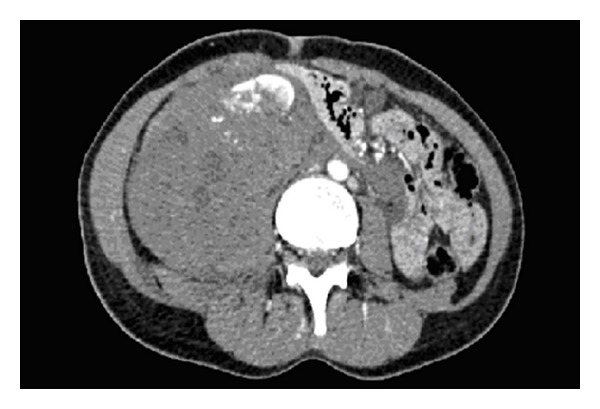


This 48-year-old female was a back seat passenger in a road traffic accident. Initial treatment consisted of resuscitation in accordance with ATLS protocol [2]. She remained haemodynamically unstable despite resuscitation with IV crystalloid and packed red cells. Urgent CT revealed ongoing right renal haemorrhage (Figures 1 and 2). The patient went to emergency theatre and was treated with lifesaving right nephrectomy via midline laparotomy. 

## 5. Patient 2 

**Figure 3:**
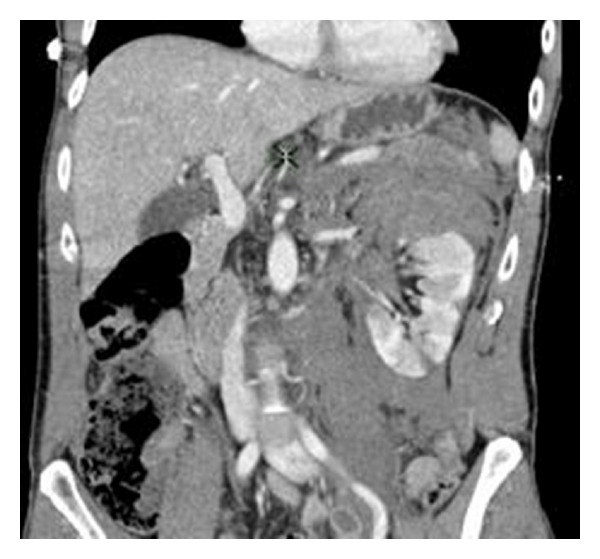


**Figure 4:**
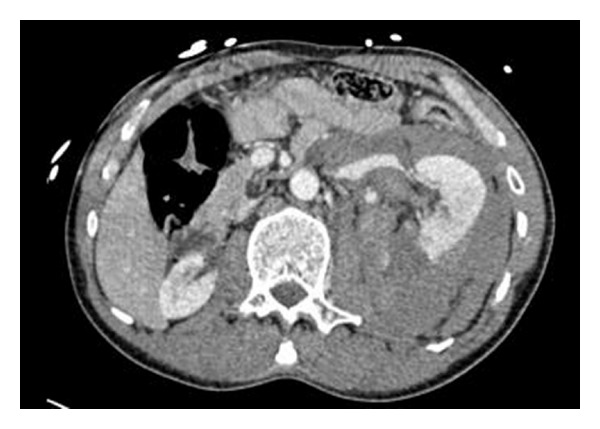


A 47-year-old man who sustained a high-impact injury whilst sledging on a mountain had ongoing left loin pain and gross haematuria on admission. CT scanning revealed a Grade 4 renal injury on the left side and a congenitally atrophic right kidney (Figures 3 and 4). He was, however, haemodynamically stable. He was managed conservatively on Surgical HDU and subsequently on the urology ward with fluids and blood products as required. 

## 6. Patient 3 

**Figure 5:**
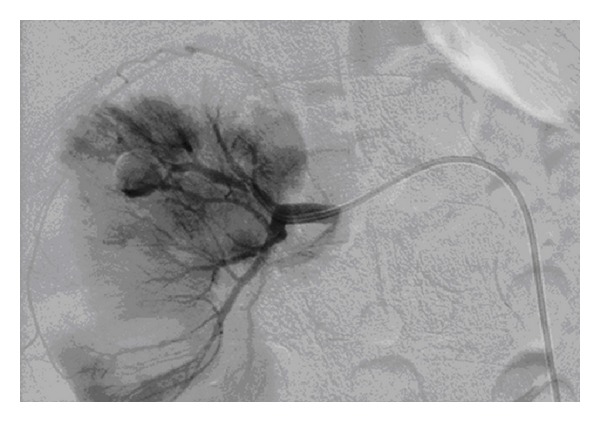


**Figure 6:**
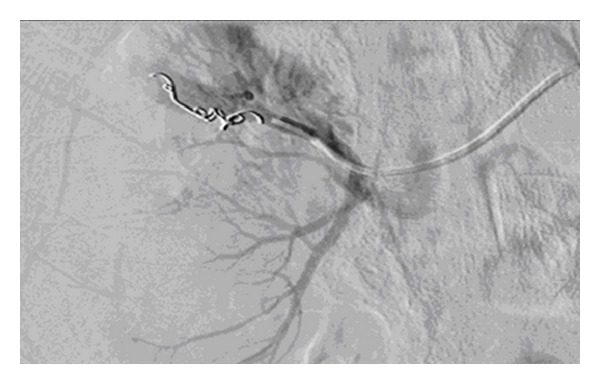


This patient aged 18 was involved in a road traffic accident and sustained a Grade 4 right renal injury. He was initially treated conservatively. However, he remained persistently tachycardic and had ongoing flank pain, and his haematuria did not settle. CT angiography revealed a right renal artery pseudoaneurysm (Figure 5), which was subsequently embolised (Figure 6). The patient felt so well after the procedure that he discharged himself against medical advice. 

## 7. Complications

 Both immediate and long-term complications of high-grade renal trauma can be significant and are likely to require followup after discharge from the ward. 

Common associated complications of renal trauma include infection, urine leak, loss of renal function, and hypertension. Urinary tract infection is the most prevalent amongst such patients managed in intensive care, and in certain cases perinephric abscess may develop. There is a niche for a randomised clinical trial to assess the use of antimicrobials in the treatment of patients with urinary leak. Most urinomas will require no treatment, as they will become absorbed, but may require drainage if persistent. Renal trauma-induced hypertension arises from the stimulation of the renin-angiotensin-aldosterone system. This can be as a result of renal arterial injury, haematoma, or arteriovenous fistula formation, which all lead to increased renin production due to diminished renal perfusion [14]. 

This case series illustrates various surgical, radiological, and conservative approaches to the management of significant blunt renal trauma, which is potentially life threatening. Most cases are managed conservatively but may require intervention immediately, or in the following days after injury. A stepwise approach and high index of suspicion in patients with higher-grade injuries who fail to progress with conservative management are required to avoid adverse outcome and morbidity. 

## Figures and Tables

**Table 1:** AAST classification of renal injuries [9].

Grade	Type of Injury	Description
I	Normal contusion	Microscopic or gross hematuria with normal urologic findings
	Hematoma	Nonexpanding subcapsular hematomas with no laceration

II	Hematoma	Nonexpanding perinephric (perirenal) hematomas confined to the retroperitoneum
	Laceration	Superficial cortical lacerations less than 1 cm in depth without collecting system injury

III	Laceration	Renal lacerations greater than 1 cm in depth without collecting system injury

IV	Laceration	Renal lacerations extending through the renal cortex, medulla, and collecting system
	Vascular injury	Injuries involving the main renal artery or vein with contained hematoma, segmental infarctions without associated lacerations

V	Laceration	Shattered kidney, ureteropelvic junction avulsions
	Vascular injury	Complete laceration (avulsion) or thrombosis of the main renal artery or vein that devascularizes the kidney
